# Long-Term Consequences of Childhood Maltreatment Among Postpartum Women—Prevalence of Psychosocial Risk Factors for Child Welfare: An Independent Replication Study

**DOI:** 10.3389/fpsyt.2022.836077

**Published:** 2022-03-14

**Authors:** Melissa Hitzler, Alexandra M. Bach, Franziska Köhler-Dauner, Harald Gündel, Iris-Tatjana Kolassa

**Affiliations:** ^1^Clinical and Biological Psychology, Institute of Psychology and Education, Ulm University, Ulm, Germany; ^2^Department of Child and Adolescent Psychiatry and Psychotherapy, University Hospital Ulm, Ulm, Germany; ^3^Department of Psychosomatic Medicine and Psychotherapy, University Hospital Ulm, Ulm, Germany

**Keywords:** childhood maltreatment, psychosocial risk factors, mental health, child welfare, prevalence, screening, postpartum

## Abstract

**Introduction:**

As an especially burdensome experience, childhood maltreatment (CM) can have lifelong consequences on the mental health and wellbeing of an individual well into adulthood. We have previously reported that CM constitutes a central risk factor not only for the development of mental problems, but also for facing additional psychosocial risks, endangering healthy development of mother and offspring throughout life (e.g., financial problems, intimate partner violence, substance use). This study was designed to replicate these findings in a larger, independent study cohort.

**Method:**

In this cross-sectional replication study an independent cohort of 533 healthy postpartum women was interviewed within seven days after parturition. CM experiences were assessed retrospectively using the German version of the Childhood Trauma Questionnaire (CTQ) and current psychosocial risk factors for child welfare were assessed using the Konstanzer Index (KINDEX).

**Results:**

Of all women, 16.1% experienced emotional and 10.1% physical abuse, 28.5% emotional neglect, 9.4% physical neglect and 10.3% experienced sexual abuse. Most importantly, the higher the CM load the more psychosocial stressors existed in women's life. In Particular, women with higher CM load had a higher risk for mental health problems, intimate partner violence, financial problems, and a higher postnatal stress load.

**Conclusions:**

In an independent sample, this study replicated the previous findings that CM and psychosocial risk factors for child welfare were strongly associated in a dose-response manner. Our results emphasize the higher vulnerability of women with a CM history in the postpartum period. To avoid negative consequences for mother and child, a regular and evidence-based screening for CM and psychosocial risk factors during pregnancy and puerperium is needed to identify at-risk mothers early during pregnancy and to provide appropriate support. Hence, our findings highlight the mandatory requirement for an interdisciplinary collaboration of gynecological practices, hospitals and midwifes, along with psychologists and psychotherapists and child and youth welfare services.

## Introduction

Childhood maltreatment (CM), i.e., experiencing sexual abuse as well as physical and emotional abuse or neglect, is considered a major psychological, psychosocial, and biological risk factor for wellbeing and health (1) and a major public health problem (2, 3). According to large scale representative studies a significant proportion of up to 47 % of adults have experienced at least one type of CM at some point during their childhood or adolescence (4–10). This can elicit detrimental sequelae for wellbeing, development and health across the whole lifespan: CM experiences are associated with an altered functioning of the biological stress-response systems [e.g., (11–13)], poorer physical and mental health and disadvantageous socio-economic and psychosocial outcomes (e.g., conflicted relationships, high stress level, poor attachment styles) which persist throughout life (14, 15). Moreover, CM experiences contribute to an increased vulnerability for the development of psychological and/or physical diseases when encountering another intense or traumatic stressor later in life (i.e., a second hit) compared to individuals without a history of CM (16, 17). Therefore, it is crucial to investigate the long-term effects of CM among at-risk groups. Postpartum women present a particularly susceptible group, as the birth of a child and the first few months postpartum are challenging and demanding. Mothers need to adapt to the new role as a mother, face bodily changes, sleep deprivation, a high workload, altered (intimate) relationships, and are possibly reminded of their own (more or less adverse) childhood. As a result of these physical and emotional stressors, the risk for mental health problems is increased in postpartum women (18, 19).

Adding to the generally higher risk for psychopathologies in the postpartum phase, studies indicate that postpartum women with a history of CM show additional stressors and risk factors (20, 21) like more relationship conflicts (22, 23), higher stress levels and less social support (24, 25). This exacerbates mothers' stress load and further increases the risk for mental health problems (25). Importantly, a higher burden of stress among mothers with a history of CM not only impairs the mothers' wellbeing and health, it also presents a risk factor for their newborns' wellbeing, as a high burden of stress and a history of CM can lead to an insecure, disorganized mother-child attachment and a general feeling of being overwhelmed by child-care. Both can negatively influence the maternal parenting style, the relationship to the child and the care for the child (26–29). Hence, a maternal history of CM can generate a disadvantageous developmental environment for the child: Children of parents with a history of CM are more likely to be victimized themselves (9, 30, 31) and suffer more often from developmental and mental health problems during childhood, adolescence and well into adulthood (26, 28, 32).

This association, however, is far from deterministic. The high variability in the consequences of CM and the comparably low transmission rates of CM (31) highlight the importance of identifying further risk and resilience factors among mothers and their children that can influence their wellbeing and health independently of CM. Indeed, several psychosocial risk factors have been identified which increase the risk for negative outcomes in the exposed mothers as well as their offspring. Prior work highlights inter- and intrapersonal risk factors like intimate partner violence or relationship conflicts, high stress levels, insecure or disorganized attachment styles, current or prior mental health problems, that can mitigate negative effects on the attachment between mother and child and the overall family context (26, 33–36). On a socioeconomic level, young parenthood, financial problems, or poor living conditions and low education can further increase this vulnerability (21, 31, 37, 38). Interestingly, these vulnerability factors seem more prevalent in women with a history of CM (20). However, living in a stable relationship, having reliable relationships, or perceiving social and emotional support can protect both mothers and their children, and can help maintaining wellbeing and fostering healthy development (26, 35, 39, 40). In line with most of the afore discussed studies, our group previously reported a significant association between several psychosocial risk factors and the severity of CM experiences in mothers shortly after giving birth [see (20)]. Specifically, the more CM had been experienced by mothers, the more psychosocial risk factors for mother's wellbeing and child welfare were present. The study by Koenig et al. (20) was one of the first to assess potential psychosocial risk factors in healthy postpartum women with a range of different severities of CM experiences. As the link of a CM history and psychosocial risk factors for child welfare can entail far reaching implications for those affected this previously found association warrants replication in other samples.

Attaining a clear and reliable understanding of which psychosocial characteristics can put mothers' and children's wellbeing and health at risk is essential for the early and accurate identification of at-risk-families and accordingly integrating them in a supportive social network and providing them with supportive and/or preventive interventions to protect mothers' and their children's wellbeing. To reach this long-term goal, valid and reliable empirical evidence for the consequences of CM is highly needed to establish screening systems and specific support networks on an empirically sound foundation. The heterogeneity in the field of CM research (e.g., methodology of the studies, definition of CM and associated risk factors, assessment, recall biases) and accordingly heterogenic findings further emphasize the need for the replication and testing of previously identified risk factors, consequences and prevalence rates of CM. Thus, this study aimed at replicating the findings by Koenig et al. (20) in a larger and independent sample of healthy postpartum women with varying degrees of CM using the same study design, recruitment strategies, and data analysis, determining the prevalence rates of maternal childhood maltreatment experiences, establishing the prevalence rates of psychosocial risk factors for maternal well-being and child welfare and testing the hypothesis that higher CM load would be associated with a higher burden of psychosocial risk factors among a community sample of healthy mothers giving birth to a child.

## Materials and Methods

### Participants and Study Design

**Figure 1:**
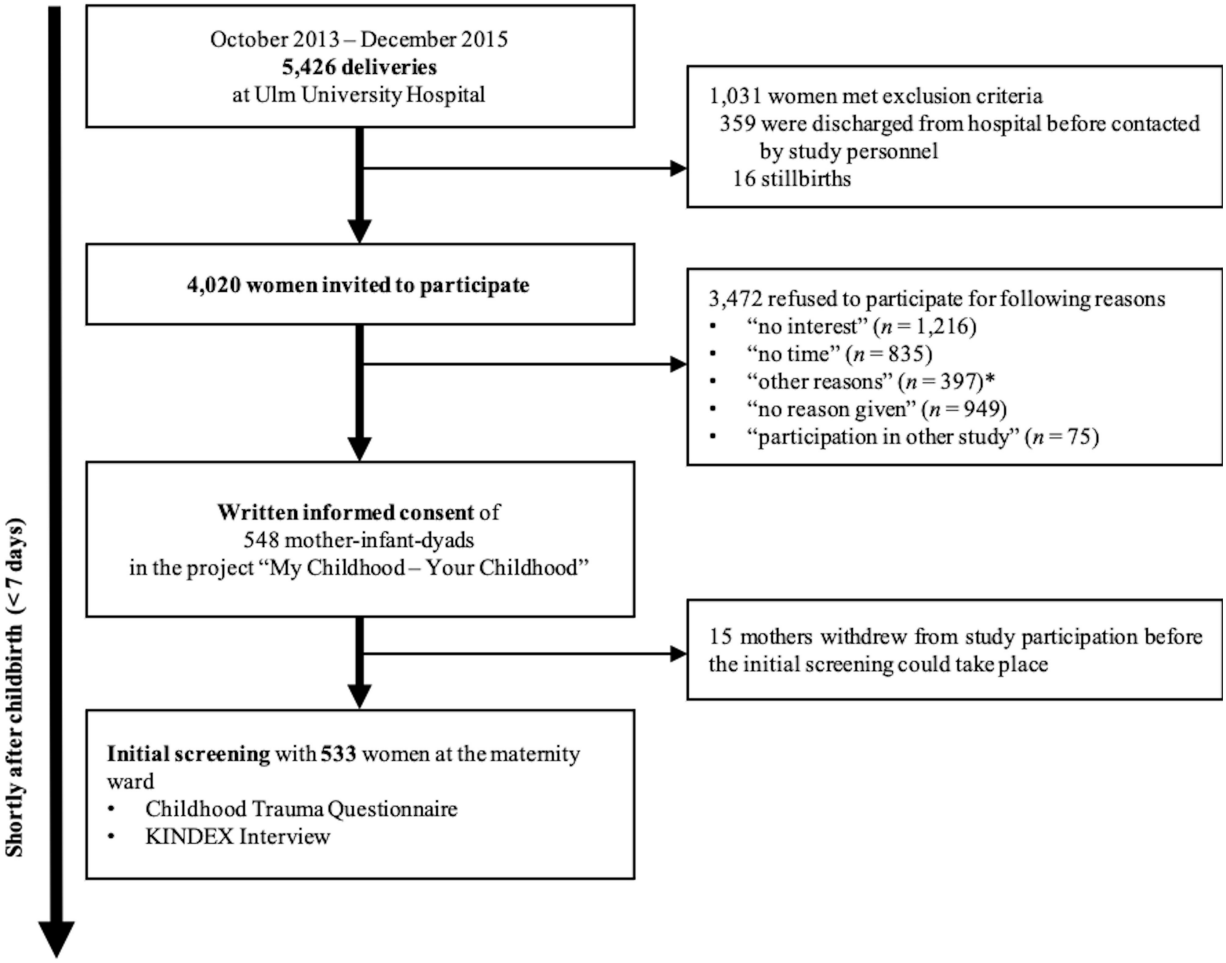
Study- flow, recruitment and withdrawal rates. *Other reasons given for participation refusal. Including: husbands not agreeing to participation of their wives, questions were too intimate, or women not wanting to talk about their childhood.

The data were collected within the project “My Childhood—your Childhood” (Oct 2013 – Dec 2016) that was designed to investigate risk and resilience factors in the intergenerational transmission of CM in a community sample of mother-infant-dyads. Women were approached in the maternity ward of Ulm University Hospital shortly after parturition (on average 2.7 days [*SD* = 4.8] postpartum). In total 548 mothers received a complete description of the study and provided written informed consent. Shortly thereafter 15 mothers withdrew their consent (see Figure 1 for detailed description of study flow and drop-out rates). Exclusion criteria for mothers were age under 18 years, insufficient knowledge of the German language, maternal psychotic disorder or acute substance abuse disorder. For ethical reasons no mother with severe complications during parturition and/or severe health problems of the child (e.g., child on neonatal intensive care unit; preterm birth ≤ 37 weeks; birth weight ≤ 2,700 g; painful/severe perineal laceration or blood loss) were approached, as well as women whose own health problems were regarded by medical staff as too severe to allow interviewing. All study procedures were approved by the Ethics Committee of Ulm University and followed the current Declaration of Helsinki.

Healthy postpartum women were interviewed by trained study personnel within 1 week after parturition. All interviewer were qualified psychologists and/or psychotherapists, who were trained and supervised by experienced psychotherapists and clinicians in trauma-sensitive psychological interview skills. Besides asking for socio-demographic and clinical characteristics, psychosocial risk factors were assessed, using the *Konstanzer Index* [KINDEX; Schauer, unpublished^1^]. The German version of the *Childhood Trauma Questionnaire* [CTQ; (41)] was used to retrospectively assess the mothers' history of CM experiences before the age of 18. To acknowledge the special emotional condition of women in the puerperium the psychological assessment was conducted as an interview. Thus, study personnel were able to intervene in case of emotional distress due to intimate questions. See Figure 1 for a detailed description of the study design.

### Childhood Maltreatment Experiences

The German version of the CTQ (41) was used to retrospectively assess the exposure to CM. Experiences of emotional and physical abuse, emotional neglect, physical neglect, and sexual abuse are assessed on a 5-point Likert scale with five items per scale (subscale sum score range 5-25). The CTQ was performed as an interview, i.e., study personnel read the questions to participants in a standardized way. Women answered according to the CTQ Likert scale, which they were given in paper form. The CTQ sum score (possible range: 25–125) provides a cumulative measure of the severity of CM experiences, here referred to as “CM load.” Established cutoff criteria subdivided the severity of each CTQ subscale into the categories “none,” “mild,” “moderate,” and “severe.” Applied were the mild cutoff criteria which classifies an individual with at least mild CM experiences as “having experienced CM” and the moderate cutoff which classifies an individual with at least moderate CM experiences as “having experienced CM.”

### Psychosocial Risk Factors for Child Welfare

To assess mothers' psychosocial risk factors, the KINDEX (42)^1^ was applied. Originally developed to help detecting and supporting high-risk women in the gynecological field, we modified the KINDEX for retrospective acquisition of psychosocial data in the postnatal period. With 31 items in total, the KINDEX covers a wide range of risk areas (11 in total) that can endanger healthy child development and mothers' wellbeing (cf. Table 1). To avoid redundancy of questions regarding CM experiences the KINDEX factor “traumatic experiences during childhood” referring to physical abuse and sexual abuse was replaced by the respective CTQ subscales. Hence, we report not 11, but 10 psychosocial risk factors of the KINDEX in the current study. Summing up all KINDEX items a cumulative sum score (possible range 0–29) can be derived. The multidimensional instrument has no built-in cutoff.

**Table 1:** Psychosocial risk factors (KINDEX) in the *N* = 533 participating mothers.

		***n*_RISK_**	**%**
1. Maternal young age	▸ ≤ 21 years	9	1.7
2. Migration	▸ Mother	84	15.8
	▸ Father	76	14.3
3. Single parent/parents living apart	▸ Mother doesn't live with child's father	12	2.3
4. Financial problems	▸ Worried about financial situation	28	5.3
	▸ Precarious housing situation^a^	7	1.3
5. Medical problems	▸ Physical complaints^b, ♠^	302	62
	▸ Pregnancy complications^c^	161	30.3
	▸ Medical risk factors^d^	121	22.8
6. Complicated prenatal attachment	▸ Unplanned pregnancy	80	15
	▸ mother's joy level low^e^	0	0
	▸ father's joy level low^e^	78	14.7
	▸ Mother's concerns high^f, †^	2	0.4
	▸ Father's concerns high^f, †^	91	17.3
7. Perceived stress levels	▸ In previous 4 weeks very high^g, ♦^	4	0.8
8. Intimate partner violence	▸ Escalating conflicts^‡^	108	20.5
	▸ Loud arguments^†^	103	19.5
	▸ Bodily conflict^†^	1	0.2
	▸ Violent partnership ever^‡^	49	9.3
9. Substance abuse	▸ Mother smokes	39	7.3
	▸ Mother consumes alcohol	56	10.5
	▸ Mother uses drugs	1	0.2
	▸ Father smokes^♦^	133	25.1
	▸ Father consumes alcohol^†^	217	41.2
	▸ Father uses drugs^‡^	7	1.3
10. Maternal Mental Illness (current or previous)	▸ Diagnosis (lifteme/current)^h^	120	22.6
	▸ Psychotropic drugs (lifetime)	105	19.8
	▸ Out-patient treatment (lifetime)	203	38.2
	▸ In-patient treatment (lifetime)	36	6.8

a *Housing index (rooms/no. of people) ≤ 0.5*.

b *E.g., birth-related symptoms (e.g., afterpains, lower abdominal/pelvic pain etc.)*.

c *E.g., breech presentation, pregnancy-related diabetes, antepartum hemorrhage*.

d *E.g., advanced maternal age, hypertension and obesity*.

e *Low = 0–3 on a scale of 0–10*.

f *High = 7–10 on a scale of 0–10*.

g *Assessed using the Perceived Stress Scale 4 (PSS-4); PSS-4 sum score ≥ 12*.

h *Diagnosis as reported by mothers: n = 62 major depressive disorder; n = 35 anxiety disorder; n = 59 other mental disorder (listed in descending frequency: Burnout/Adjustment disorder; eating disorder; Posttraumatic Stress Disorder, Substance/Alcohol use disorder, Borderline Personality Disorder, Psychosis/Schizophrenia; Bipolar Disorder, Panic Disorder, ADHD, Somatoform disorder); Note: some mothers reported more than one mental disorder*.

♠ *n = 487*.

† *n = 527*.

‡ *n = 528*.

♦ *n = 529*.

### Statistical Analyses

Data were analyzed with R version 4.0.2 (43). The critical *p*-value for statistical significance was set to 0.05. Descriptive and correlational statistics of all variables were examined. Descriptive data were described as mean values (*M*) and standard deviations (*SD*) or absolute (*n*) and relative frequency (in %). Relationships between CM load and psychosocial risk factors of the KINDEX were calculated using Spearman's rank correlations (*r*_s_) for ordinally scaled data. Correlations between each CTQ subscale were calculated with Spearman's rank correlations due to skewed data. Due to the replicational character of the current study we refrained from *p*-value correction for multiple comparisons. Because of the theoretical overlap between the KINDEX and the CTQ, the KINDEX risk area “traumatic experiences during childhood” was removed from the analysis of the association between CM (CTQ sum score) and psychosocial risk factors (KINDEX sum score) to avoid multicollinearity.

## Results

### Demographics and Clinical Characteristics

On average, participating postpartum mothers were 32.4 years of age (*SD* = 4.6 years; range = 18–44 years). For 247 mothers (46.4%) the current pregnancy and for 286 mothers (54.6%) the current parturition was the first one. The mean number of children (incl. the newborn) was 1.6 (*SD* = 0.8) ranging from 1 to 6 children. About two thirds of the women (68.1%; *n* = 362) had achieved a general higher education level (cf. A-Level) and more than a half had graduated from university (57.1%; *n* = 304). 84.2% of the women (*n* = 448) and 85.2% (*n* = 453) of the fathers were of German origin. Almost all women lived in a committed relationship (98.5%; *n* = 524) at the time of birth. 76.1% (*n* = 404) of them were married. 95.9% (*n* = 510) cohabitated with their romantic partner. An overview of the sample characteristics can be found in Table 2.

**Table 2:** Sociodemographic and clinical characteristics of *N* = 533 postpartum women.

	**%**
**Sociodemographics**	
German origin^a^	84.2
Higher academic education	68.1
Currently employed	89.3
Committed relationship	98.5
Cohabitation with romantic partner	95.9
**Pregnancy and parenthood**	
Planned pregnancy^‡^	85
Caesarean section^♠^	27.2
Former abort^§^	6.2
Former miscarriage^#^	22.3
Maternal caretaker own mother	98.7
Maternal caretaker own father	91.9
Separation own parents^♦^	27
**Clinical characteristics**	
Complication during current pregnancy^*^	30.3
Acute pain/physical complaints^†^	56.7
Medical risk factors (e.g., diabetes, overweight, high blood pressure)^†^	22.8
Mental disorder lifetime	22.6
Psychotropic medication lifetime	19.8
Chronic physical disease^♦^	35.5

*^a^Non-German origins: other European countries (N = 47), Russia (N = 13), Kazakhstan (N = 10), Brazil (N = 3), other countries with one occurrence each (Afghanistan, Argentina, Cape Verde, Cuba, Indonesia, Iran, Kenia, Cuba, Morocco, Switzerland, Tadzhikistan, USA)*.

‡ *n = 502*.

# *n = 520*.

♦ *n = 529*.

♠ *n = 526*.

§ *n = 517*.

* *n = 402*.

† *n = 487*.

### Prevalence of Childhood Maltreatment

CM load ranged from 25 to 96 points (*M* = 32.74; *SD* = 11.57). Overall, 196 of 533 women (36.8%) reported mild to severe CM experiences in at least one subtype of CM. 102 of 533 women (19.1%) experienced more than one type of maltreatment. Applying a stricter cutoff criterion for CM (i.e., having experienced at least moderate levels of CM), 111 women (20.8%) were classified as having experienced at least one form of moderate to severe CM during their childhood and 42 (7.9%) of all women had experienced more than one type of moderate to severe maltreatment.

For the different subtypes of maltreatment, the following pattern occurred (cf. Table 3): At least mild e*motional neglect* was observed in 152 women (28.5%), at least moderate in 59 women (11.1%). At least mild *physical neglect* was present in 50 women (9.4%), at least moderate in 22 women (4.1%). At least mild *emotional abuse* was seen in 86 women (16.1%), at least moderate in 36 women (6.8%). At least mild *physical abuse* had been experienced by 54 women (10.1%), at least moderate by 30 women (5.6%). Fifty-five women reported at least mild *sexual abuse* (10.3%), at least moderate was present in 46 women (7.1%). All types of abuse and neglect correlated highly significant between each other (all ranging from *r*_*s*_ = 0.172 to *r*_*s*_ = 0.591, all *p* < 0.01).

**Table 3:** CM prevalence rates in % of current replication study compared to Koenig et al. (20), Moody et al. (44), Stoltenborgh et al. (8), Prevoo et al. (10), and Witt et al. (5, 6).

	CM cutoff^*^	***n***	**Age range in years (*M; SD*)**	**CM total**	**Emotional neglect**	**Physical neglect**	**Emotional abuse**	**Physical abuse**	**Sexual abuse**
**Current Study** ^**1**^	mild	533	18–44 (32.4; 4.6)	36.8	28.5	9.4	16.1	10.1	10.3
	moderate				11.1	4.1	6.8	5.6	7.1
Koenig et al. (20)^1^	mild	240	21–46 (33.1; 5.2)	40.4	32.1	7.5	13.8	6.7	12.5
Moody et al. (44)^2^	-	-	-	-	27.0^5^		21.7	12.2	13.2
Stoltenborgh et al. (8)^2^	-	-	-	-	18.4	6.5	29.2	22.9	13.5
Prevoo et al. (10)^2^	-	-	-	-	18.4	16.3	36.3	22.6	18.0^3^
Witt et al. (6)^4^	moderate	2510	14–94 (48.4; 18.2)	31.0	13.3	22.5	6.5	6.7	7.6
Witt et al. (5)	-	2531	> 14 (48.6; 18.0)	43.7	13.4	4.3	12.5	9.1	4.3

* *CM Cutoffs differed between studies. Applied cutoffs are reported where they were explicitly indicated. Cutoffs in the current study, in Koenig et al. (20) and Witt et al. (6) based on the established cutoff criterion of the CTQ (41). The mild cutoff classifies an individual with at least mild CM experiences and the moderate cutoff classifies an individual with at least moderate CM experiences as “having experienced CM.”*

1 *The sample consisted of women only*.

2 *Systematic Review of CM prevalence rates in different continents—as prevalence rates differ markedly between countries, only data for Europe is presented here*.

3 *Prevalence rates for sexual abuse refer to a sample of girls only. Prevalence rates for boys are significantly lower with 7.4%*.

4 *Reported are prevalence rates for at least moderate levels of CM*.

5 *The authors reported only a combined measure of neglect experiences*.

### CM and Psychosocial Risk Factors

The frequencies of all individual psychosocial risk factors reported as assessed by the KINDEX are displayed in Table 1. The three most frequent psychosocial risk areas were *maternal medical problems, intimate partner violence* and current or previous *maternal mental illness*. The psychosocial risk sum score of all postpartum women in the current sample ranged from 0 to 17 and was on average 4.38 (*SD* = 2.64).

**Figure 2:**
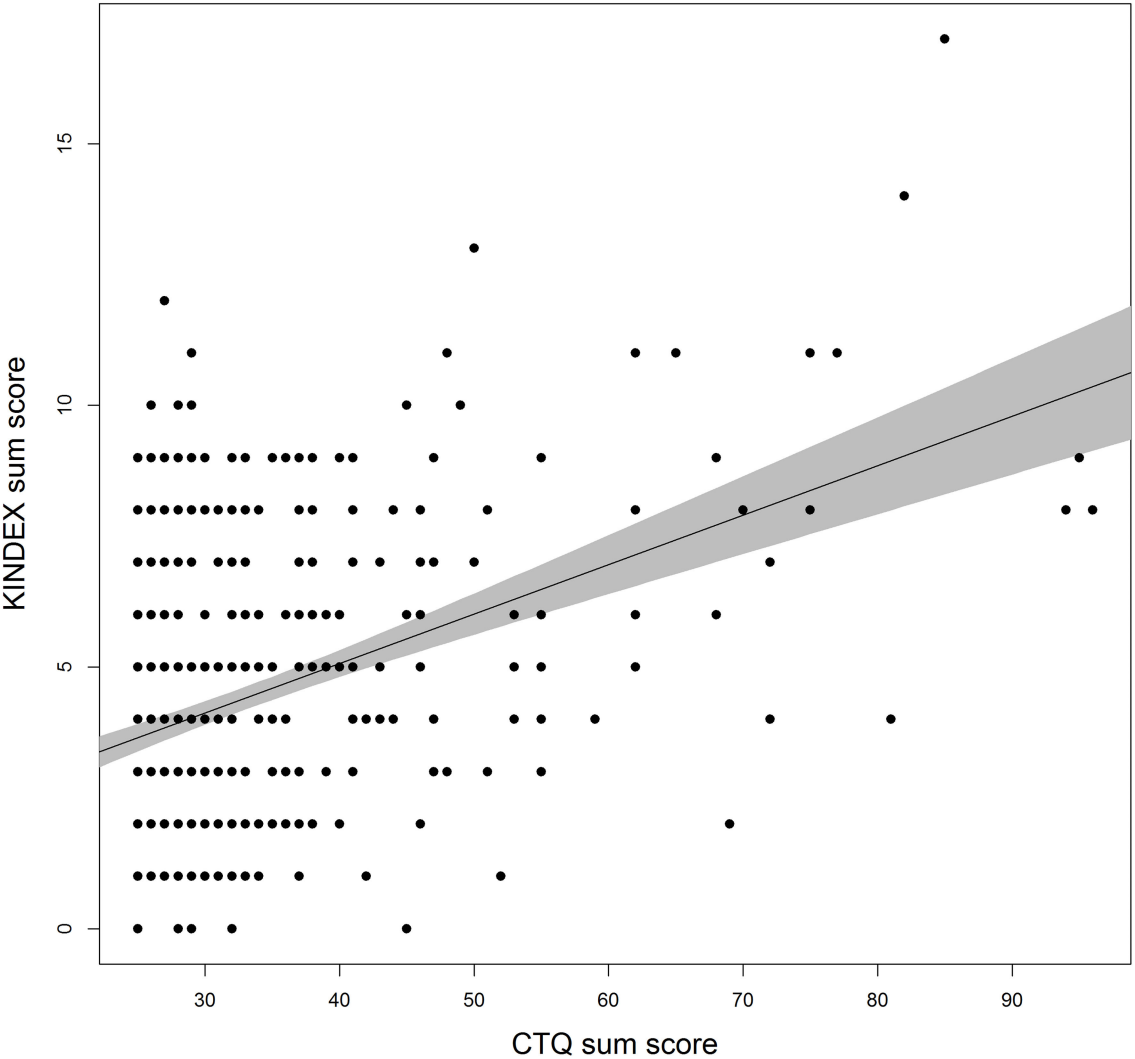
Estimated linear association between CTQ and KINDEX sum scores. Bullets represent raw data points. The gray area depicts the 95% confidence interval. Possible range of CTQ [25; 125]. Possible range of KINDEX sum score [0; 29].

The associations of CM load with the different KINDEX risk factors are shown in Table 4. CM load correlated significantly with the psychosocial risk factors on the KINDEX shortly after parturition (*r*_*s*_ = 0.34, *p* < 0.001; see Figure 2). CM load correlated most strongly with self-reported maternal mental illness (*r*_*s*_ = 0.35, *p* < 0.001), intimate partner violence (*r*_*s*_ = 0.27, *p* < 0.001), perceived stress levels (*r*_*s*_ = 0.13, *p* ≤ 0.001), and medical (*r*_*s*_ = 0.13, *p* ≤ 0.001) and financial problems (*r*_*s*_ = 0.13, *p* ≤ 0.001).

**Table 4:** Spearman's rank correlation of KINDEX risk factors with maltreatment load (represented by the CTQ sum score) of *N* = 533 postpartum women.

**KINDEX risk factor**	***r_***s***_***	***p***
Maternal young age	0.04	0.15
Migration	0.08	0.04^*^
Single parent	0.02	0.33
Financial problems	0.13	<0.001^***^
Medical problems	0.13	<0.001^***^
Complicated prenatal attachment	0.08	0.03*
Very high perceived stress level^a^	0.13	<0.001^***^
Intimate partner violence	0.27	<0.001^***^
Substance abuse	0.08	0.04*
(Previous) maternal mental illness	0.35	<0.001^***^

* *p < 0.05*,

*** *p < 0.001*.

a *Assessed using the Perceived Stress Scale 4 (PSS-4) sum score*.

## Discussion

The current study replicated our previous findings of an increased psychosocial risk in postpartum women with a history of CM (20). The dose-response relationship between psychosocial risk factors and CM load could be confirmed: the higher the CM load that had been experienced, the more psychosocial risk factors for child welfare were present at the transition to parenthood. The results of this independent replication further emphasize the serious long-term consequences of CM across the whole lifespan: Our results suggest that affected postpartum women seem to be faced with more psychosocial risk factors and thus may be more vulnerable when they are exposed to stressors and demanding situations later in life than unaffected individuals. Psychosocial risk factors in the postpartum phase constitute serious risk factors in the etiology of mental health problems and an overall diminished wellbeing in postpartum women—in particular for those with a history of CM. This not only impairs the women's wellbeing but endangers the children's welfare and healthy development at the same time.

Prevalence rates for CM reported here in a community sample of healthy postpartum women are widely consistent with previously described prevalence rates in Germany and Europe including our own pilot study [cf. Table 3; for details see [(5, 6, 8, 10, 20, 44)]. Physical neglect, however, seemed underrepresented in our sample with prevalence rates of 9.4% (at least mild CM) respectively 4.1% (at least moderate CM) compared to other studies [see (7) prevalence between 22.5 and 28.8% for at least moderate physical neglect]. This is in line with the findings of our previous pilot study where physical neglect and physical abuse were similarly underrepresented compared to other studies [e.g., (6, 7)]. One reason for this discrepancy might be due to the comparatively small age range and the generally younger mean age of the postpartum women in both studies compared to other representative studies [e.g., age range of 14–94 years in Witt et al. (6)]. Especially studies in German speaking countries found evidence that the older the participants in German samples, the more physical abuse and neglect had been reported due to experiences during the post-second-world war period (food shortage, more corporal punishment) in Germany and due to a disparate parenting style during this time (4, 45). Another reason for the comparably low rates of physical neglect in the current sample is presumably the relatively good regional socioeconomic status of participating mothers (e.g., high education, low unemployment rate, stable financial situation) which can be a compensating factor the consequences of CM (15). Other contributing factors may include study design and method, sample characteristics and different definitions of CM [see (10)]. Nevertheless, with percentages of 36.8% of at least mild CM and 20.8% of at least moderate CM, an alarming proportion of women examined in our healthy community sample were directly affected by CM. Our findings therefore emphasize the significance of CM as a serious social and public health problem which affects individuals of all socioeconomic and social classes.

However, the central finding of the current study is the substantial amount of psychosocial risk factors the mothers faced at the transition to parenthood. Most importantly, CM load and psychosocial risk factors for child welfare were positively associated in a dose-response relationship. This is in line with our previous study and with a large body of evidence, highlighting the negative psychosocial long-term consequences of CM across the lifespan [e.g., (14, 20, 33)]. As central risk factors, we identified and replicated more “*intimate partner violence”* and more maternal (acute or prior) “*mental health problems”* as well as a higher “*perceived stress level,” “medical problems,”* and “*financial problems”* to be particularly strong associated with CM load. A concatenation of risk factors that can create an environment which endangers wellbeing and health in the affected women and their children equally. Simultaneously, this implies a lack of familial protective resources to buffer negative effects of CM and other stressors in the postpartum phase. Indeed, along with a higher risk for intimate relationship conflicts (46), women with a CM history perceived less social support from their romantic partner in the postpartum phase (25). Support from a romantic partner, however, reduces not only stress but protects mental health in postpartum women (25, 47) and can even buffer the effects of CM on the mother-child interaction (39, 48). As a result, and in accordance with the stress-sensitization hypotheses (16), CM-affected postpartum women exhibit an even higher risk for stress-related psychopathologies, including (postpartum) depression and anxiety (17, 49–51) compared to unaffected mothers, as infant-related stressors, CM history and a higher load of psychosocial risk factors cumulate.

From an intergenerational perspective a maternal history of CM and/or the above mentioned psychosocial risk factors can lead to a disadvantageous family environment and maladaptive parenting style: Lower parenting competence, less maternal involvement, higher infant-related stress load, an impaired mother-child-attachment, insensitive, inconsistent and harsh parenting behavior or even hostility and rejection toward the child are associated with maternal psychosocial risk factors and CM (29, 33, 52). A concatenation that can put healthy child development at risk. Studies show that on a parental level these factors even may increase the risk for children towards a disadvantageous development or to experience CM themselves (53, 54). As aforementioned, this association is far from deterministic and transmission rates of CM are comparatively low (31). While a history of CM certainly is a risk for negative health outcomes for mother and child, the high variability in the consequences of CM highlight the impact of further psychosocial risk and resilience factors that can have an influence on the family environment and children's wellbeing and health.

To counteract psychosocial risks on maternal and intergenerational level, at-risk women (having experienced CM and/or facing psychosocial risk factors) and their children should be identified and referred to appropriate prevention programs early during pregnancy and in the postpartum phase. Screening methods for psychosocial risk factors and CM, like the KINDEX (42) as one example, can help to identify at-risk individuals, preferably before the child is born. This allows referral to intervention programs to prepare the parents for the postpartum phase, to protect them from negative outcomes and to promote their resilience. Promisingly, supportive and preventive intervention concepts have been established [see (20, 55–58)]. First efforts were made to train and qualify health and social professionals to recognize at-risk families and provide early practical assistance for them [e.g., (56)]. Early interventions for families and children have already proven to be effective in supporting at-risk families and promoting caregiving and child development by helping them to compensate e.g., psychosocial disadvantages, high stress load, intimate partner conflicts or the consequences of CM (55, 57–61).

Despite CM and associated psychosocial risk factors being a prevalent and consequential major social as well as public health problem with far reaching intergenerational consequences the assessment system is still insufficient and little is known about its true prevalence rates [see (7, 45)]. Prevalence rates of CM considerably vary due to differences in CM definition, assessment methods, study design and types of maltreatment (10). Therefore, a precise, comprehensive, empirically tested and evidence-based assessment system of CM and psychosocial risk factors can help to make important steps toward child protection and the access to early intervention and support networks for at-risk individuals. However, systematic screening for CM and psychosocial risk factors is still an enormous, controversially discussed challenge [see: (45, 58, 60, 62–64)]. Screening tools must be based on empirically tested predictive factors to appropriately and accurately identify persons at need. Identification of such factors will enable professionals to target and provide services to those children at risk. However, in the context of CM and psychosocial risk factors there are still different definitions, assessment strategies and methods which make systematic, reliable, and strategic screenings for CM and psychosocial risk factors difficult (10, 45, 60, 63, 64) and research on established screening methods and their application to practice is still needed (60). Moreover, while it is of great importance to assess CM and psychosocial risk factors for preventive measures, researchers, clinicians, social services, and policy makers must be aware that psychosocial risk factors for child welfare and CM experiences are very sensitive information. When discussing them, including their possible consequences, one should be careful not to evoke unintended negative effects like stigmatization or stereotype-driven attitudes toward at-risk families (60, 62). Again, a history of CM and psychosocial risk factors are not deterministic for maladaptive parental behavior or environments. Another controversially discussed point is: A systematic screening for CM and psychosocial risk factors is neither justified, appropriate nor ethical, when there is no effective or available and accessible intervention or support [for a critical review see: (62, 63)]. Finally, it seems clear that screening for risk factors in the postpartum phase that impair women's health and child development yields great potential for affected individuals. The ongoing challenge is to figure out how to do it appropriately and to establish evidence-based, accessible, and effective support networks for at-risk families (60, 63). In the end the question remains: How to identify and consequently provide service to those women and families at-risk?

Some limitations of the study need to be mentioned. The study sample consisted of relatively healthy postpartum women, living mostly in a committed relationship, with high education level and good socioeconomic status who reported mostly mild to moderate CM experiences. This should be kept in mind when applying the results to other cohorts and contexts as the generalizability might be limited. Due to the study design, the study's subject and the applied methods, one can assume that women with severe or even traumatic CM experiences and a higher load of psychosocial risk factors may have refrained from participating in the study—even if participants didn't state this explicitly as a reason. As we assessed CM retrospectively, we cannot completely rule out that factors like mood, method (retrospective self-assessment), misinterpretation, memory, mental disorders or subsequent life events influenced the quality of the CM recall and therefore our prevalence rate. However, recent studies suggest that recall bias only had a minimal influence on the association of CM and psychopathology or other risk factors and didn't invalidate study results (64–66). Additionally, the assessment of CM in an interview could have influenced the answering pattern of the postpartum women to give more socially acceptable answers. Therefore, we assume that CM load, CM prevalence rates and psychosocial risk factors for child welfare were rather underestimated in our sample. However, the association between CM load and psychosocial risk factors in our healthy community sample demonstrates the deleterious long-term effects of CM and further psychosocial risk factors and underlines the need for screening and preventive measures—even in individuals with mild CM and a relatively high socioeconomic status. Mothers with a history of CM have a higher risk to show an unfavorable constellation of psychosocial risk factors, however, this does not imply that mothers without a history of CM can't show a similar constellation of psychosocial risk factors. Therefore, screening for a wide range of evidence-based psychosocial risk factors, in addition to CM, seems the more comprehensive way to identify at-risk families.

## Conclusion

Experiencing maltreatment and neglect during childhood and adolescence is associated with a wide range of psychosocial risk factors for health and wellbeing, as well as for the overall healthy development of affected individuals. Especially in times of increased stress and demand, like the postpartum phase, CM-affected women are especially vulnerable to further stressors and at an even higher risk to experience further psychosocial risk factors and negative outcomes for their health and wellbeing. This not only impairs the women but can put their children's welfare and healthy development at risk, too. As CM experiences and their intergenerational transmission are a serious and consequential issue, it is mandatory to improve accessible and individually tailored and transdisciplinary support by social services, health professionals and policy makers. Hence, a valid and reliable screening for psychosocial risk factors for childhood maltreatment is an important first step to identify at risk families. However, to protect at-risk families from stigmatization and negative outcomes due to the screening, these assessments must be implemented within a professional framework where outcomes are treated carefully, it must be voluntary, evidence-based, ethically justifiable. Moreover, after identification support must be available and accessible. As there is still no common definition or methodology to assess CM and psychosocial risk factors, this is still an important interdisciplinary field of research with important implications for researchers, practitioners, and policy makers.

## Data Availability Statement

The datasets for this manuscript are not publicly available because the data may not be passed on or published to third parties outside the research project. The dataset contains sensitive personal and clinical information that might allow identifying individual participants. We do not have the ethics committee's or our participants' consent to grant access to the collected data. Requests to access the datasets should be directed to the corresponding author.

## Ethics Statement

The studies involving human participants were reviewed and approved by Ulm University Ethics Committee. The patients/participants provided their written informed consent to participate in this study.

## Author Contributions

I-TK and HG developed the study concept and study design. AMB collected clinical data with the support of MH. MH performed statistical analyses, interpreted the data, and drafted the manuscript. All authors read and approved the final manuscript.

## Funding

The Federal Ministry of Education and Research provided funding for data collection (Grant Number: 01KR1304A). MH and AMB were supported by a PhD scholarship of the Konrad-Adenauer-Foundation.

## Conflict of Interest

The authors declare that the research was conducted in the absence of any commercial or financial relationships that could be construed as a potential conflict of interest.

## Publisher's Note

All claims expressed in this article are solely those of the authors and do not necessarily represent those of their affiliated organizations, or those of the publisher, the editors and the reviewers. Any product that may be evaluated in this article, or claim that may be made by its manufacturer, is not guaranteed or endorsed by the publisher.

## Footnotes

^1^Schauer M, Ruf-Leuschner M. *KINDEX: Pränatale Erfassung psychosozialer Belastungen als Risiken für die Entwicklung über die Lebensspanne - der Konstanzer* INDEX (2016).
